# The clinical significance of Psoriasin for non-small cell lung cancer patients and its biological impact on lung cancer cell functions

**DOI:** 10.1186/1471-2407-12-588

**Published:** 2012-12-10

**Authors:** Mu Hu, Lin Ye, Fiona Ruge, Xiuyi Zhi, Lijian Zhang, Wen G Jiang

**Affiliations:** 1 Cardiff University-Capital Medical University Joint Centre for Biomedical Research, Cardiff, UK; 2 Metastasis & Angiogenesis Research Group, Cardiff University School of Medicine, Cardiff, CF14 4XN, UK; 3 Lung Cancer laboratory & Department of Thoracic Surgery, Xuanwu Hospital, Capital Medical University, Beijing, 100053, P.R. China; 4 Department of Thoracic Surgery, Peking University School of Oncology and Beijing Cancer Hospital & Institute, Beijing, 100142, P.R. China

**Keywords:** Psoriasin, S100A7, Lung cancer, Adhesion and invasion

## Abstract

**Background:**

Psoriasin (S100A7) is a member of the S100 gene family. Alteration of Psoriasin expression has previously been reported to play an important role in cancer aggressive behaviour. The current study sought to investigate the level of Psoriasin expression at the mRNA level in a cohort of patients with non-small cell lung cancer (NSCLC), the association with clinical implication and outcomes, and the molecular and cellular impact of the protein on lung cancer cells.

**Methods:**

Fresh frozen NSCLC cell carcinoma tissues, along with matched normal tissues were obtained from 83 NSCLC patients who received curative resection from January 2003 to December 2011. The expression of Psoriasin in the NSCLC specimens was assessed using both quantitative real time PCR (QPCR) and immunochemical staining. Knockdown and forced expression of Psoriasin in NSCLC cell lines were carried out using constructed plasmid vectors carrying either ribozyme transgenes targeting human Psoriasin or full-length coding sequence, respectively. The effect of Psoriasin on the functions of NSCLC cells was determined using a variety of *in vitro* cell function assays.

**Results:**

Higher mRNA levels of Psoriasin were observed in tumour tissues when compared to both the paired normal background tissues and none paired normal tissues (p = 0.0251 and 0.0195). The mRNA level of Psoriasin was found to be higher in the squamous carcinoma (P=0.035). Higher Psoriasin expression is associated with poor prognosis. The cell function tests had supportive results to the clinical findings. Over-expression of Posriasin in lung cancer cells (SK-MES-1) resulted in an increase in *in vitro* growth and invasiveness. In contrast, Psoriasin knockdown suppressed cell growth and invasion (P<0.05), but increased cell adhesion (P<0.05).

**Conclusions:**

Psoriasin expression is increased in lung cancer, more specifically in lung squamous carcinoma compared with adenocarcinoma, and is associated with poor prognosis. Psoriasin plays crucial roles in regulating the growth and invasion of lung cancer cells.

## Background

Non-small cell lung cancer (NSCLC) is highly aggressive. Better prognosis and clinical outcomes mainly rely on early detection and curative resection. However, some of these early stage patients have poor prognosis due to metastasis. Real-time-quantitative-PCR-based assay of certain genes may help to identify patients of early-stage NSCLC with higher risks of poorer prognosis [1]. Psoriasin, also known as S100A7, is a member of the S100 gene family that was identified as a 11.4 kDa protein induced in the epidermis isolated from psoriasis [2]. The *Psoriasin* gene maps to chromosome 1q21.2-q22, within a region that encompasses at least 12 of the S100 gene family and several other epidermal differentiation genes [3]. Other members of the S100 calcium binding proteins have been implicated in a range of biological processes, including tumour metastasis [4]. Alteration of Psoriasin (S100A7) expression has previously been reported to play an important role in cancer aggressive behaviour [5]. Psoriasin is likely to be one of those molecules associated with the development of the invasive phenotype and the transition from preinvasive to invasive in breast cancer with the capability for subsequent metastasis associated with poor outcome in oestrogen receptor-negative invasive breast cancer [6]. While Kesting *et al.* reported that Psoriasin is a positive marker for oral carcinogenesis and early tumour progression [7]; recent studies have however shown that down-regulation of Psoriasin in ER negative breast cancer cells inhibits EGF-induced migration [8]. Psoriasin has also been shown to enhance tumour growth in ER negative cells by regulating prosurvival mechanisms, such as NF-κB and AKT pathways [9]. Morgan *et al.* identified a novel interaction between Psoriasin and β6-integrin and demonstrated that it was required for αVβ6-integrin dependent invasion by cancer cells. Inhibition of this interaction may represent a novel therapeutic strategy to target carcinoma invasion [10]. Evidence of Psoriasin having a role in lung cancer is sparse. It has been recently shown that Psoriasin expression was associated with brain metastases of lung squamous carcinoma and may be a potential biomarker [11]. However, function of Psoriasin in NSCLC remains unknown.

The current study sought to investigate the level of expression of Psoriasin at the mRNA level in a group of surgical patients with NSCLC and to examine the association of this molecule with clinical features and outcomes. We also provide new insights into the biological functions of Psoriasin in NSCLC cell lines.

## Methods

### Cell lines and human lung cancer specimens

Human lung squamous carcinoma SK-MES-1 and human lung adenocarcinoma A549 cells were obtained from the American Type Culture Collection (ATCC, Manassas, VA, USA). Cells were routinely cultured with Dulbecco’s modified Eagle medium (DMEM) supplemented with 10% foetal calf serum, penicillin and streptomycin (Gibco BRC, Paisley, Scotland, UK). Fresh frozen NSCLC cell carcinoma tissues at TNM stages of I to IIIa, along with matched normal tissues, were obtained from 83 patients who received curative resection in Peking University Cancer Hospital and Xuanwu Hospital of Capital Medical University from January 2003 to December 2011. Ethical approval was provided by both Peking University Cancer Hospital and Xuanwu Hospital of Capital Medical University Ethics Committees. Clinical information of the patients is given in Table 1. These tissues were collected immediately after surgical resection and stored in the Tissue Bank of Peking University Oncology School and Xuanwu Hospital Lung Cancer Laboratory. Clinic-pathological factors, including age, sex, histological types of tumours, TNM stage, and lymph node metastasis were recorded and stored in the patients’ database. Patients were followed up from the day of operation to December 2011. The follow-up intervals were calculated as survival intervals after surgery.

**Table 1:** Psoriasin expression and clinical/pathological characteristics of lung cancer

**Clinical/pathological features**	**Sample no.**	**Median (Copy no.)**	**IQR**	**P-value**
**Tumour tissue**	83	0.17	0-27.5	
**Background tissue**	69	0.01	0-1	**0.0195**
**Paired tumour tissue**	61	0.17	0-24.1	
**Paired background tissue**	61	0.01	0-1	**0.0251**
**Gender**				
**Male**	47			
**Female**	25			
**Histology**				
**Squamous carcinoma**	30	0.4	0-45	
**Adenocarcinoma**	37	0.1	0-4.1	**0.035**
**Others**	6	N/A	N/A	
**TNM staging**				
**I**	22	0	0-15.3	
**II**	16	0.1	0-110.8	0.22
**III**	34	1	0-43	0.085
**Tumour differentiation**				
**High**	5	0.1	0-27.2	
**Med**	33	1	0-42	0.17
**Low**	11	0.01	0-1.15	0.51
**Tumour status**				
**T1**	8	3.99	0-13.87	
**T2**	41	0	0-22.2	0.989
**T3**	23	25.6	0-225.6	0.081
**Lymph node status**				
**N0**	24	0	0-18	
**N1**	13	0.1	0-77.6	0.93
**N2**	25	1	0-42	0.51
**Smoking status**				
**Former or current smoker**	49	0.2	0-43	
**Non smoker**	23	0	0-12	**0.0372**

### RNA isolation and reverse transcription polymerase chain reaction

Total RNA was isolated from the homogenized NSCLC tissues (83 pairs of specimens) and cell lines using Total RNA Isolation Reagent (ABgene™). Reverse transcription was performed using the Reverse Transcription kit (Primer design), followed by PCR using a REDTaq™ ReadyMix PCR reaction mix (Sigma-Aldrich, Inc.). The quality of DNA was verified using GAPDH primers (sense: 5’-ATGATATCGCCGCGCTCGTC-3’; antisense: 5’-CGCTCGGTGAGGATCTTCA-3’). Psoriasin mRNA levels were assessed using Psoriasin primers as follow: F: 5-GGGCACAAATTACCTCGCGA, R: 5-CACTGGCTGCCCCCGGAAC. PCR was performed in a GeneAmp PCR system 2400 thermocycler (Perkin-Elmer, Norwalk CT, USA). Cycling conditions for the 16-μl-reaction mixture were 30s at 94°C for denaturation, 30s at 55°C for annealing and 30s at 72°C for elongation (30 cycles). This was followed by a final 10 min extension period at 72°C. PCR products were then separated on a 2% agarose gel. The product was then visualized under ultraviolet light following ethidium bromide staining.

### Quantitative real time PCR (QPCR)

QPCR was performed on the Icycler IQ5 system (Bio-Rad, Hammel Hemstead, UK) to quantify the level of Psoriasin transcripts in the NSCLC specimens (shown as copies/μl from internal standard). NSCLC cDNA samples were then examined for Psoriasin transcript expression, along with a set of standards and negative controls. The QPCR technique utilised the Amplifluor system™ (Intergen Inc., England) and QPCR master mix (BioRad). Pairs of primers were designed using Beacon Design software (PREMIER Biosoft, Palo Alto, CA): Psoriasin QPCR primers: ZF: 5-TGTGACAAAAAGGGCACAAA, ZR: 5-ACTGAACCTGACCGTACACCCAGCAAGGACAGAAACTC. The underlined sequence in the reverse primers was the additional Z sequence, which is complementary to the universal Z probe (TCS Biologicals Ltd., Oxford, UK). Real-time QPCR conditions were 95°C for 15 min, followed by 60 cycles of 95°C for 20 s, 55°C for 30 s and 72°C for 20 s. QPCR for GAPDH was also performed on the same samples to normalize for any residual differences in the initial level of RNA in the specimens, using a GAPDH quantitation kit from Perkin-Elmers (Perkin-Elmer, Surrey, England, UK).

### Immunohistochemical staining of psoriasin

Paraffin sections of NSCLC (n = 16) and paired normal lung tissues (n = 16) were cut at a thickness of 6 μm. The sections were first dewaxed using a series of xylene and rehydrated through descending grades of ethanol washes. Endogenous peroxidase activity was blocked with 0.3% hydrogen peroxide for 15 min. For antigen retrieval, sections were boiled in 10 mM citrate buffer (pH 6.0) for 10 min. The sections were then immersed in TBS wash buffer for 10 min to rehydrate and incubated for 20 min in a horse serum blocking solution before probing with the Psoriasin antibody (1:100) (ab13680, Abcam, Cambridge, UK) together with a negative control without primary antibody. Following extensive washing, sections were incubated for 30 min with the secondary biotinylated antibody (Vector Laboratories). Avidin-biotin complex (Vector Laboratories) was then applied to the sections for 30 min followed by extensive washing. Diamino benzidine chromogen (Vector Laboratories) was then added to the sections and incubated in the dark for 10 min. Sections were then counterstained in Mayer’s haematoxylin and dehydrated in ascending grades of ethanol before clearing in xylene and mounting under a cover slip. Staining was independently assessed by the authors.

### Construction of Psoriasin expressing and ribozyme transgenes, and transfection

The full sequence of Psoriasin was amplified using the standard PCR procedure described above and a master mix with proof reading enzyme, as previously reported [12,13]. The following primers were used for amplification of the full length human Psoriasin: sense primers, 5’- ATGAGCAACACTCAAGCTG; antisense: 5’- ACTGGCTGCCCCCGGAACA. Correctly amplified product was then cloned into pEF6/V5-His-TOPO vector (Invitrogen, Paisley, UK). Multiple clones of E. coli were screened and plasmids from the clones were sequenced. Purified plasmids were then electroporated into the SK-MES-1 cell line. Blasticidin (5 μg/ml final concentration) was used to select stably transfected strains. The control group of cells containing the same plasmid vector (minus the Psoriasin sequence) was termed SK-MES-1 PEF. Anti-Psoriasin ribozyme transgenes were employed to knockdown the expression of Psoriasin in the A549 cell line, and were generated using the methods previously described [12]. Briefly, the anti-Psoriasin hammerhead ribozyme was designed based on the secondary structure generated using Zuker’s RNA mFold program. Then the ribozymes that specifically target Psoriasin were generated using touchdown PCR with the appropriate primers (sense, 5’-CTGCAGTCACAGGCACTAAGGAAGTTGGGCTGATGAGTCCGTGAGGA; antisense, 5’-ACTAGTGGCTGGTGTTTGACATTTCGTCCTCACGGACT). The constructed ribozyme trangenes were transfected into A549 cells by way of electroporation. A549 cells transfected with anti-Psoriasin ribozyme and the control cells transfected with the empty plasmid vectors were designated as A549 PsoRib and A549 PEF respectively.

### Western blotting

To detect the expression level of FAK in the NSCLC cell lines, confluent cells were pelleted and then lysed using a lysis buffer containing 2.4 mg/ml Tris, 4.4 mg/ml NaCl, 5 mg/ml sodium deoxycholate, 20μg/ml sodium azide, 1.5% Triton, 100 μg/ml PMSF, 1 μg/ml leupeptin, and 1 μg/ml aprotinin, for 45 min at 4°C. After lysis and centrifugation at 13,000 rpm for 15min, protein concentration of each sample was measured using an improved Lowary assay (DC Protein Assay kit, Bio-Rad). The samples were adjusted to equal concentrations with sample buffer and then boiled at 100°C for 5 min, before separation on a 10% polyacrylamide gel. Following electrophoresis, these separated proteins were blotted onto nitrocellulose membrane and blocked in 10% skimmed milk (w/v in TBS) for 1 hour. The membranes were then probed with the anti-phosphorylated FAK at Tyrosine residue 407 (Santa Cruz, CA, USA) and anti-GAPDH-antibody (Santa-Cruz Biotechnologies, California, USA) as internal control, followed by a peroxidase-conjugated secondary antibody (1:1,000). Protein bands were visualised using an ECL system (Amersham, UK), and photographed using an UVITech imager (UVITech, Inc.).

### *In vitro* cell growth assay

Cells were plated into 96-well plates at density of 2,000 cells/well. The cells were then fixed in 4% formaldehyde after 1, 3 and 5 days respectively. 0.5% crystal violet (w/v) was used to stain cells. Following washing, the stained crystal violet was dissolved with 10% (v/v) acetic acid and the absorbance was determined at a wavelength of 540 nm using a spectrophotometer (Bio-Tek, ELx800). The absorbance of each cell line at day3 and day5 were then normalised against day1 absorbance.

### Cell matrix adhesion assay

The cell matrix adhesion assay was done as previously described [14]. A 96-well plate was precoated with 5 μg of Matrigel and allowed to dry. Following rehydration by serum free medium, 20,000 cells were added to each well, and treated with or without 200nM FAK inhibitor (FP573228, Tocris, Bristol, UK). After 45 min of incubation non-adherent cells were washed off using BSS buffer. The remaining cells were fixed with 4% formalin and stained with 0.5% crystal violet. Following washing, the stained crystal violet was dissolved with 10% (v/v) acetic acid and the absorbance was determined at a wavelength of 540 nm using a spectrophotometer (Bio-Tek, ELx800).

### Wounding/migration assay

The wounding assay was performed as previously described [15]. The cells were seeded at a density of 25,000 per well into a 24-well plate and allowed to reach confluence. The monolayer of cells was then scraped with a fine gauge needle to create a wound. The movement of cells to close the wound was recorded as described previously using a time-lapsed video system. Images were captured from the videotape at the equivalent of 15 min intervals in real-time and stored as a series of gray scale bitmap images. The movement of single cells within a colony was analyzed by tracking each cells boundary, for each frame in a series, using the Optimas 6.0 motion analysis (Meyer Instruments, Houston, Texas).

### *In vitro* invasion assay

Transwell inserts (upper chamber) with 8 μm pore size were coated with 50 μg of Matrigel (Collaborative Research Products, Bedford, Massachusetts, USA) and air-dried. Following rehydration, cells were seeded at a density of 20,000 per insert and allowed to invade for 3 days. After incubation, cells that had migrated through the matrix and adhered to the other side of the inserts were fixed in 4% formalin, stained with 0.5% (weight/volume) crystal violet, and counted under a microscope.

### Statistical analysis

Statistical analysis was performed using MINITAB version 13.32 (Minitab Inc., State College, PA). The relationship between Psoriasin expression and tumour grade, TNM staging and nodal status was assessed by Mann–Whitney U test. The error bars shown in the graphs represent the STDEV. Survival was analyzed using Kaplan-Meier survival analysis. Differences were considered statistically significant at p < 0.05.

## Results

### The expression of Psoriasin mRNA and protein in NSCLC tissues

Psoriasin transcript expression was examined in the lung specimens of 83 NSCLC patients using real-time quantitative PCR (Table 1) (expressed as mean Psoriasin transcript copies/μl of RNA from 50 ng total RNA and standardized with GAPDH). Higher mRNA expression levels of Psoriasin were observed in tumour tissues p=0.0251 and p=0.0195 when compared to the paired normal background tissues and unpaired normal tissues, respectively.

**Figure 1:**
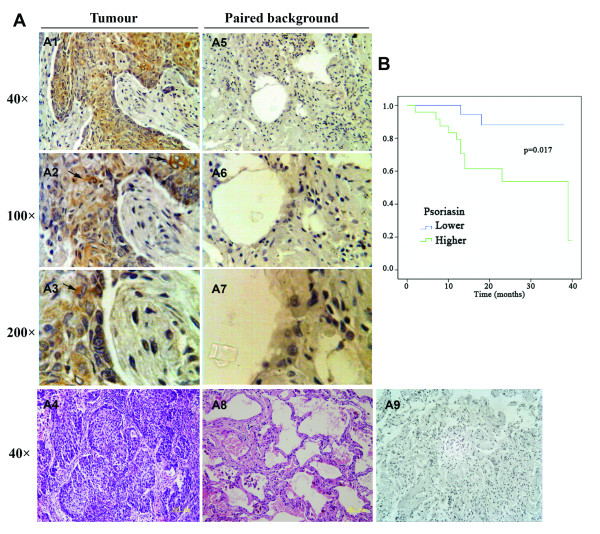
**Increased expression of Psoriasin in human lung cancer.A**, Expression of Psoriasin in squamous carcinoma (A1 to A3) and paired background tissues (A5-A7). Staining of Psoriasin in tumour cells is indicated by arrows. A4 and A8 are matched HE staining for the squamous carcinoma specimen and background tissue. A9 is the negative control of IHC using the secondary antibody alone. **B**, Psoriasin expression and overall survival of lung cancer.

To assess the expression pattern of Psoriasin at the protein level, we performed immunohistochemical analysis of Psoriasin in the paired human NSCLC and normal tissue sections, using a specific anti-Psoriasin monoclonal antibody (n = 16). Psoriasin was almost absent from normal tissues and adenocarcinoma tumours. However, it was interesting to note that squamous carcinoma tissues had a highly positive staining of Psoriasin, mostly in the cytoplasmic region of the tumour cells (Figure 1).

### The association of Psoriasin expression with clinical and histopathological features of NSCLC

The relation of Psoriasin expression to specific pathological status was assessed in the present study. In comparison with its expression in adenocarcinoma tissues, higher levels of Psoriasin transcripts were seen in the squamous carcinoma tissues (p = 0.035). The patients who were former or current smokers had a higher Psoriasin expression compared with the patients who did not smoke at all (p=0.0372).

The relationships between Psoriasin expression and clinical TNM staging and Lymph node status were also analyzed. Statistical analysis showed no significant difference among different groups (Table 1). An average of Psoriasin transcript levels in the tumours of TNM stage 2 was used as a threshold between the high and low expression. Kaplan-Meier analysis showed poorer overall survival in the patients with higher expression levels of Psoriasin, p=0.017 compared with the patients that had lower expression of Psoriasin (Figure 1B). The average survival of lower Psoriasin expression patients was 34.4 months (95%CI, 30.9-37.8 months), while that of patient with higher expression was 27.7 months (95%CI, 20.6-34.8 months).

### Creation of sublines of lung cancer cells with psoriasin over-expression and knockdown

**Figure 2:**
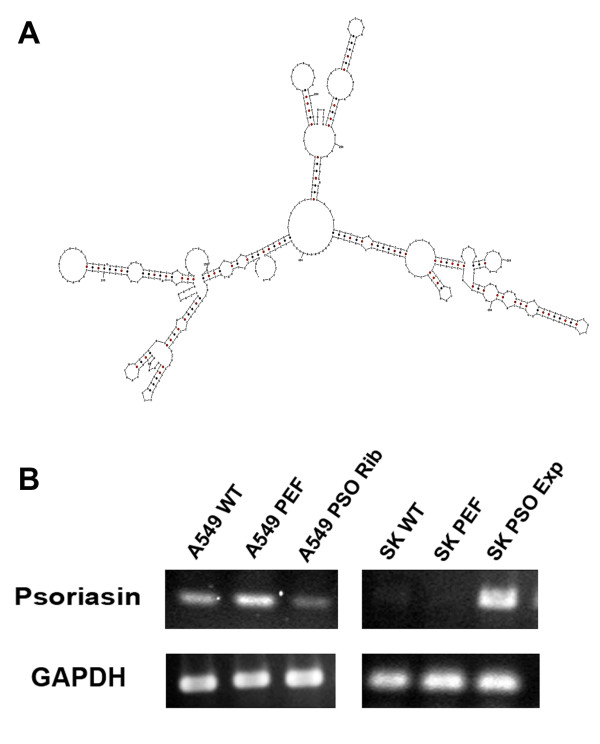
**Knockdown and forcing expression of Psoriasin in NSCLC cells.A**, Secondary structure of Psoriason mRNA which was used to design anti-Psoriasin ribozymes. **B**, Establishment of SK-MES-1 cells for expressing Psoriasin and A549 cells for Psoriasin knockdown were verified using RT-PCR.

A panel of NSCLC cancer cell lines was examined for the presence of Psoriasin using RT-PCR. Psoriasin transcript was detectable in A549 cell lines, but not expressed in the SK-MES-1 cell lines (Figure 2). To investigate the role of Psoriasin in NSCLC cells, we used SK-MES-1 for Psoriasin over-expression, and A549 cells for knockdown of Psoriasin using an anti-Psoriasin transgene (based on the secondary structure of Psoriasin mRNA, Figure 2A). Psoriasin over-expression was successfully established in SK-MES-1 cells (SK-MES-1 PsoExp) after transfection compared with that in SK-MES-1 WT (SK-MES-1-wild-type) and empty vector control (SK-MES-1 PEF) cells (Figure 2B). Psoriasin presenting in the wild type (A549 WT) and empty vector control cells (A549 PEF) cells was reduced in the A549 Psoriasin knockdown cells (A549 PsoRib). These Psoriasin modified sublines were used for the following *in vitro* studies.

## Effects of Psoriasin over-expression and knockdown on *in vitro* growth of NSCLC cells

**Figure 3:**
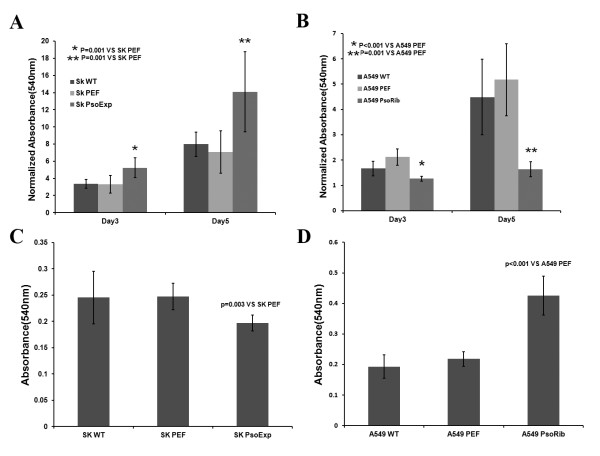
**Influences on*in vitro*growth and adhesion of NSCLC cells by Psoriasin over-expression and knockdown.A**, Growth of SK-MES-1 PsoExp cells was increased compared to SK-MES-1 PEF control at day3 and day5 (P=0.001 and P=0.001 respectively). **B**, A549 PsoRib cells exhibited reduced growth over 3 days and 5 days culture, P<0.001 and P=0.001 compared to A549 PEF control, respectively. **C**, Cell adhesion in SK-MES-1 PsoExp cells was decreased (p=0.003). **D**, A549 PsoRib cells had a remarkable increase in cell adhesion (P<0.001).

We first determined the effect of Psoriasin over-expression on *in vitro* cell growth. An increase was seen in the growth of SK-MES-1 cells of Psoriasin overexpression. SK-MES-1 PsoExp NSCLC cells had a significantly increased rate of growth, p=0.001 compared to the controls (Figure 3A). This was consistent with observations in A549 PsoRib cells, in which Psoriasin expression had been knocked down and a decreased growth was seen, p < 0.001 compared to the control groups (Figure 3B).

### Impact on *in vitro* cell matrix adhesion by Psoriasin over-expression or knockdown

**Figure 4:**
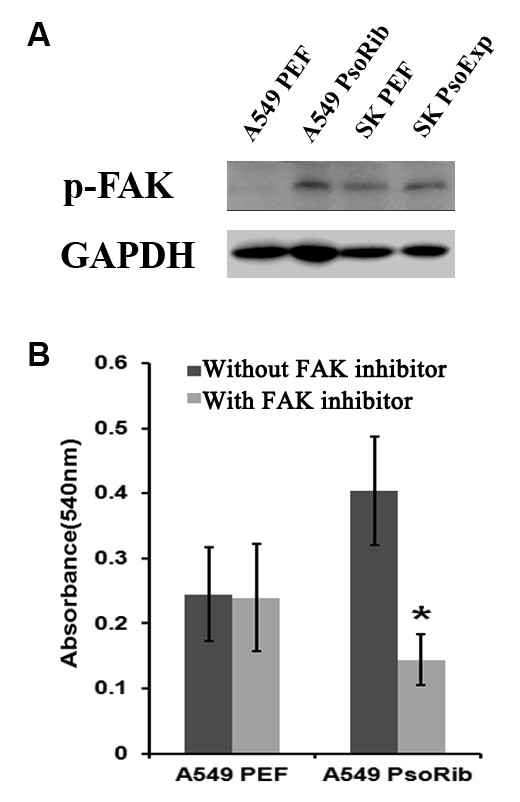
**Involvement of Focal Adhesion Kinase (FAK) in adhesion of NSCLC cells affected by Psoriasin.A**, An increased phosphorylation of FAK was seen in A549 cells after knockdown of Psoriasin, while no obvious effect was seen in the SK PsoExp cells. **B**, Addition of 200nM FAK inhibitor diminished the effect of Psoriasin knockdown on adhesion of A549 cells. * indicates p<0.01 v.s. controls.

We further examined the influence of Psoriasin on the adhesive nature of these NSCLC cells. Over-expressing Psoriasin in SK-MES-1 significantly reduced the adhesive properties compared to the control groups (Figure 3C). In contrast, knockdown of Psoriasin expression resulted in a remarkable increase in adhesive ability of A549 cells (Figure 3D). To investigate the pathway by which the adhesion function may be altered, we evaluated the activation of the focal adhesion kinase, FAK. Phosphorylated FAK (Tyr 407) was elevated in A549 PsoRib compared to A549 PEF control (Figure 4A). Furthermore, the increased adhesion by Psoriasin knockdown was diminished by the addition of FAK inhibitor (Figure 4B).

### Effects of Psoriasin manipulation on *in vitro* invasion

**Figure 5:**
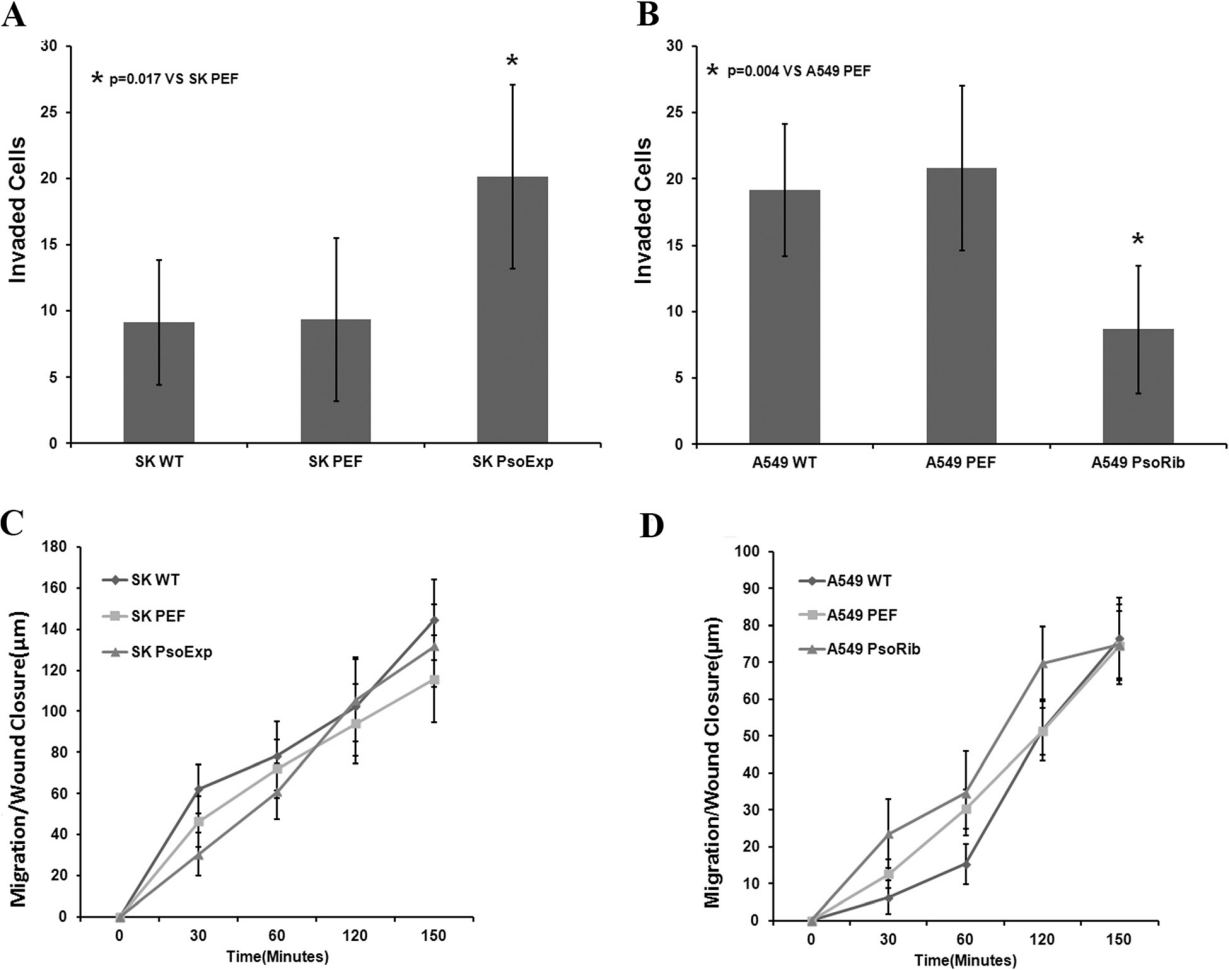
**Effect of Psoriasin knockdown and over-expression on invasion and migration of NSCLC cells.A**, Invasion was enhanced in SK-MES-1 PsoExp cells, p=0.017 compared to SK-MES-1 PEF control cells. **B**, A549 PsoRib showed a reduction of invasion, p=0.004 compared to A549 PEF control. **C** and **D**, There was no change of migration seen in cells of both Psoriasin over-expression and knockdown in comparison with the respective controls.

Over-expression of Psoriasin in SK-MES-1 resulted in a marked elevation of invasion (Figure 5A). This was also confirmed by further determination of the invasive nature of Psoriasin knockdown cells. A549 PsoRib cells were significantly less invasive than the control cells which expressed Psoriasin (Figure 5B).

### Effects of Psoriasin over-expression or knockdown on *in vitro* migration

*In vitro* wounding assay was employed to examine the influence of Psoriasin over-expression or knockdown on the migration of NSCLC cells. Over-expression of Psoriasin did not influence the migratory nature of SK-MES-1 cells (Figure 5C). The result was also consistent with observations in the Psoriasin knockdown cells, A549 PsoRib cells, where the motility was similar to that of the control cells (Figure 5D).

## Discussion

Psoriasin has been considered to be expressed in a cell type or tissue specific manner. S100A7 is over-expressed in hyperproliferative skin disease-psoriasis [2] and is also associated with the early stages or invasion of certain cancers [6,16]. Classifying a NSCLC to be an adenocarcinoma, squamous cell carcinoma or large cell carcinoma is generally difficult, especially for cancers with poorly differentiated morphologies [17-20]. Meanwhile, it has become apparent that adenocarcinoma and squamous carcinoma of the lungs have distinct mutation profiles, which underline their divergent responses to targeted therapies [21]. Therefore, identification and characterisation of biomarkers are of great value in diagnosis and therapy for specific pathological types of lung cancer. In the current study, we have shown that Psoriasin is frequently down-regulated or absent from lung adnocarcinoma cancer cells and normal lung tissues, but is often over-expressed in lung squamous carcinoma tissues. It has been reported that elevated Psoriasin protein can be detected in the sera of patients with lung squamous cell carcinomas rather than adenocarcinoma [22]. These findings may make Psoriasin an indicator for differential diagnosis for lung squamous carcinoma and adenocarcinoma. We also report that the levels of Psoriasin are correlated with the clinical outcomes and long term survival of the patients with NSCLC.

The study further demonstrates that over-expression of Psoriasin is linked to the elevation of growth, invasion and motility of NSCLC cells *in vitro*. The knockdown of Psoriasin inhibits the growth and invasion of NSCLC cells, which is also supported by findings in other malignancies [12,23]. The over-expression of Psoriasin on the other hand resulted in decreased cell adhesion while knockdown increased it. The influence of Psoriasin on cell adhesion was impaired by FAK inhibitor. Psoriasin has been shown to be up-regulated in the cells losing attachment [24]. This appears to be not just a response of cells when they lose attachment but, the expression of Psoriasin in the cells can also affect the adhesion of the cells which we have seen in our studies of Psoriasin in cell lines of different cancers. For example, in the current study, A549 cells exhibited an enhanced adhesion after the knockdown of Psoriasin which was accompanied with an increased p-FAK. It suggests that Psoriasin is inversely associated with cell adhesion in which FAK is involved. However, such mechanism was not affected in the SK-MES-1 cells of Psoriasin over-expression. It indicates that Psoriasin is a mediator or a factor involved in the adhesion, rather than a key factor or an initiating factor.

The SK-MES-1 cells did exhibit an increased invasion without obvious change in the p-FAK. In our recent study of Psoriasin in prostate cancer cells, matrix metalloproteinases (MMPs) have been indicated in the effect on invasion of cancer cells by Psoriasin [12]. After the loss of adhesion, MMPs may be consequently affected by the up-regulated Psoriasin leading to enhanced invasiveness. Although FAK has been linked to enhanced cell adhesion and migration [25,26], certainly its role in the regulation of these cellular functions appears to be more complicated as contrasting effects on the same functions by FAK have also been demonstrated [27]. Collectively these observations suggest that Psoriasin is inversely associated with the adhesiveness of lung cancer cells in which the FAK pathway may be involved. Psoriasin plays a positive role in regulation of growth and invasion of NSCLC cells.

Perhaps the most important observation seen in the present study is the association between the higher levels of Psoriasin transcripts and poor prognosis. These data clearly indicate that Psoriasin is a promoting factor in the disease progression of NSCLC and can be utilised as a potential prognostic indicator which needs to be further investigated in a larger cohort. Selective expression of Psoriasin has been shown in certain types of lung cancer, such as squamous cell carcinomas and large cell carcinomas, but its expression appears to be lower or absent from adenocarcinomas and small cell carcinomas [22]. Psoriasin has also been indicated in the brain metastasis of lung squamous cell carcinoma [11], which may account for worse prognosis. Together with the findings of the present study, it is suggested that Psoriasin has diagnostic and therapeutic value in NSCLC, particularly for lung squamous carcinoma.

## Conclusions

Taken together, the expression of Psoriasin is increased in lung squamous carcinoma compared with normal lung tissues and lung adenocarcinoma. The elevated expression is associated with poorer overall survival. Psoriasin is involved in the regulation of growth and invasion of NSCLC cells, and its expression is inversely associated with cell adhesion. These results indicate a prognostic and therapeutic potential of Psoriasin in lung cancer.

## Competing interests

The authors declare that they have no competing interests.

## Authors’ contributions

MH and LY contributed equally to the study design, experimental work, data analysis and preparation of the manuscript. XZ and WGJ contributed to the study design, data analysis and manuscript preparation. All the authors read and approved the manuscript.

## Pre-publication history

The pre-publication history for this paper can be accessed here:

http://www.biomedcentral.com/1471-2407/12/588/prepub
